# Intranasal Immunization Using Mannatide as a Novel Adjuvant for an Inactivated Influenza Vaccine and Its Adjuvant Effect Compared with MF59

**DOI:** 10.1371/journal.pone.0169501

**Published:** 2017-01-04

**Authors:** Shu-Ting Ren, Xue-Mei Zhang, Peng-Fei Sun, Li-Juan Sun, Xue Guo, Tian Tian, Jian Zhang, Qi-Yuan Guo, Xue Li, Li-Jun Guo, Jin Che, Bing Wang, Hui Zhang

**Affiliations:** 1 Department of Pathology, School of Basic Medical Sciences, Xi’an Jiaotong University Health Science Center, Xi’an, China; 2 No. 6 Vaccine Workshop, Changchun Institute of Biological Products Co., Ltd., Changchun, China; 3 Department of Pathology, Xi’an City Center Hospital, Xi’an, China; 4 Reagent R&D Dep. Scientific Research Management Center, Capital Bio Technology, Beijing, China; 5 Changchun Institute of Biological Products Co., Ltd., Changchun, China; 6 Therapeutic Vaccines Engineering Center of Shaanxi Province, Xi’an Jiaotong University Health Science Center, Xi’an, China; 7 Department of Pharmacy, Xi’an Medical University Health Science Center, Xi’an, China; University of South Dakota, UNITED STATES

## Abstract

Intranasal vaccination is more potent than parenteral injection for the prevention of influenza. However, because the poor efficiency of antigen uptake across the nasal mucosa is a key issue, immunostimulatory adjuvants are essential for intranasal vaccines. The immunomodulator mannatide or polyactin (PA) has been used for the clinical treatment of impaired immunity in China, but its adjuvant effect on an inactivated trivalent influenza vaccine (ITIV) via intranasal vaccination is unclear. To explore the adjuvant effect of PA, an inactivated trivalent influenza virus with or without PA or MF59 was instilled intranasally once a week in BALB/c mice. Humoral immunity was assessed by both the ELISA and hemagglutination inhibition (HI) methods using antigen-specific antibodies. Splenic lymphocyte proliferation and the IFN-γ level were measured to evaluate cell-mediated immunity. The post-vaccination serum HI antibody geometric mean titers (GMTs) for the H1N1 and H3N2 strains, antigen-specific serum IgG and IgA GMTs, mucosal SIgA GMT, splenic lymphocyte proliferation, and IFN-γ were significantly increased in the high-dose PA-adjuvanted vaccine group. The seroconversion rate and the mucosal response for the H3N2 strain were significantly elevated after high-dose PA administration. These adjuvant effects of high-dose PA for the influenza vaccine were comparable with those of the MF59 adjuvant, and abnormal signs or pathological changes were not found in the evaluated organs. In conclusion, PA is a novel mucosal adjuvant for intranasal vaccination with the ITIV that has safe and effective mucosal adjuvanticity in mice and successfully induces both serum and mucosal antibody responses and a cell-mediated response.

## Introduction

Seasonal influenza is a common acute respiratory viral infection that causes annual epidemics with significant morbidity and mortality in high-risk populations [1–3]. Vaccine immunization against influenza is the most effective intervention. Immunization stimulates both humoral and cellular responses and induces the production of antigen-specific antibodies, which inhibit virus attachment to target cell membrane receptors and thereby limit virus infectivity [4]. Currently, the major route of vaccination is muscular injection, which mainly induces serum IgG antibodies without inducing IgA secretion in the mucosal surfaces of the respiratory tract. Mucosal vaccination is an attractive strategy for the prevention of infection because this route evokes both systemic and local mucosal immunity to induce IgG and secretory IgA (SIgA) production [5–7]. Therefore, mucosal vaccines and their adjuvants have recently become a focus of vaccine research.

Intranasal vaccination may be more potent than parenteral injection for the prevention of influenza due to its effectiveness in preventing infection via the respiratory tract. Furthermore, this vaccination route has additional advantages; for instance, intranasal vaccination is painless, has higher acceptance for recipients, is safer, and is easily administered, which facilitates mass immunization campaigns [5, 8–11]. However, immunostimulatory adjuvants are essential for intranasal vaccines because the poor efficiency of antigen uptake across the nasal mucosa is a key issue [12]. Recently, some intranasal influenza vaccines were licensed in the United States and Switzerland, including FluMist and Nasalflu. However, these influenza vaccines can cause some side effects and are not ideal for use in high-risk populations [13–15]. Thus, it is necessary to devise alternative methods to induce mucosal immunity and to circumvent the side effects of intranasal influenza vaccines.

Mannatide, which is also known as alpha-polyactin or polyactin A (PA), was developed in China. PA is a heteropolysaccharide isolated from the fermentation broth of buccal alpha-hemolytic *Streptococcus* strain No. 33.1. PA is an immunomodulator and adjuvant that has been used for the treatment of impaired immunity, including cancer and chronic hepatitis B, in China for more than 30 years [16, 17]. A previous study showed that PA improved the production of the antibodies against hepatitis B surface antigen (anti-HBs) after hepatitis B virus vaccine immunization [18]. Therefore, we hypothesized that PA might be an ideal potential adjuvant for vaccines. Our previous study demonstrated that PA was a potential mucosal immune adjuvant that increased the immunogenicity of the H1N1 split vaccine and enterovirus 71 (EV71) whole virus inactivated antigen when administered by the intranasal vaccination route in mice [19, 20]. However, the adjuvant effect of PA for the inactivated trivalent influenza vaccine is unclear, and its safety requires further evaluation.

The adjuvant MF59 is a submicron oil-in-water emulsion that has been shown to be safe and efficient when administered intramuscularly or intranasally in humans [2]. In the present study, the immunogenicity of the PA-adjuvanted inactivated trivalent influenza vaccine was explored after intranasal vaccination in mice and compared with MF59-adjuvanted inactivated trivalent influenza vaccine to confirm the adjuvant effect of PA. Additionally, the safety of PA was investigated.

## Materials and Methods

### Animals

This study was approved by the Laboratory Animal Administration Committee of Xi’an Jiaotong University and performed according to the University Guidelines for Animal Experimentation. Specific pathogen-free female BALB/c mice aged 6–8 weeks were purchased from Xi'an Jiaotong University Laboratory Animal Centre (Xi'an, China). All mice were housed in an air-conditioned barrier system animal room at 18–22°C with 40%-60% relative humidity and a 12 h light/dark cycle and received sterilized food and filtered tap water ad libitum.

### Vaccine antigens and adjuvants

The seasonal influenza virus antigens were produced by Changchun Institute of Biological Products Co., Ltd. (Changchun, China) and were derived from embryonated eggs using production procedures followed for clinical grade influenza vaccines. The unadjuvanted inactivated trivalent influenza vaccine (ITIV) contained 150 μg of each of the three influenza hemagglutinin (HA) antigens A/NYMCX-179A (A/H1N1), A/IVR-165 (A/H3N2), and B/NYMCBX-39 (B) per 1 mL dose. The adjuvant PA was purchased from Sinopharm Chuankang Pharmaceutical Co., Ltd. (Sichuan, China). The two adjuvanted ITIVs with different PA doses contained the above unadjuvanted vaccine combined with 50 mg or 100 mg of PA per 1 mL dose. An adjuvant consisting of an MF59 oil-in-water emulsion was prepared with 5% squalene (Sigma-Aldrich, USA), 0.5% Tween 80 (Sigma-Aldrich, USA), and 0.5% Span 85 (Sigma-Aldrich, USA) in 10 mM citrate using a sonifier cell disrupter (JY92-IIN, Ningbo Xinzhi Biotech Co., Ltd., Zhejiang, China) as previously described [21]. The MF59-adjuvanted ITIV was prepared by mixing the MF59 adjuvant and the above unadjuvanted vaccine 1:1 (v/v) prior to immunization.

### Immunization and experimental design

Each mouse was immunized intranasally with the above vaccines or phosphate-buffered saline (PBS) in a 10 μL volume (5 μL per nostril) at a 1-week intervals on days 0, 7 and 14. The control animals were immunized with PBS without the HA antigens and adjuvant (Control group, n = 12). During each immunization, the mice in the unadjuvanted vaccine group (Vaccine group, n = 12) received 1.5 μg of HA antigens without any adjuvant, whereas the mice in the low-PA-adjuvanted vaccine group (LPA-V group, n = 12), high-PA-adjuvanted vaccine group (HPA-V group, n = 12) or MF59-adjuvanted vaccine group (MF59-V group, n = 12) were administered 1.5 μg of HA antigens in combination with 500 μg of PA, 1000 μg of PA or 5 μL of MF59, respectively (Table 1).

**Table 1:** Intranasal immunization design in the different groups.

Groups	n	Time	Volume/mouse*	HA/each strain/mouse*	Adjuvant/mouse*
**Control**	12	days 0, 7, and 14	10 μL	–/PBS	–/PBS
**Vaccine**	12	days 0, 7, and 14	10 μL	1.5 μg	–
**LPA-V**	12	days 0, 7, and 14	10 μL	1.5 μg	500 μg of PA
**HPA-V**	12	days 0, 7, and 14	10 μL	1.5 μg	1000 μg of PA
**MF59-V**	12	days 0, 7, and 14	10 μL	1.5 μg	5 μL of MF59

* The description of each immunization.

### Serum or nasal and trachea wash sample collection

Blood samples were collected via the angular vein on days -3 and 21 of the first vaccination. Serum samples were obtained by centrifuging the blood samples at 3000 rpm for 15 minutes and were stored at -18°C prior to use. On day 21 after the first immunization, all mice were anesthetized with ether and sacrificed, and the heads of the mice were cut at the level of the medulla. The nasal cavity and trachea were washed three times with 200 μL of PBS, centrifuged at 2000 rpm for 10 minutes and stored at -18°C prior to use.

### Enzyme-linked immunosorbent assay (ELISA) for antigen-specific serum or nasal and trachea wash antibodies

Influenza virus antigen-specific antibodies were measured by ELISA. Briefly, polystyrene 96-well plates (Nunc, China) were coated with whole ITIV antigens (HA, 1 μg/mL per strain) in 0.1 M bicarbonate buffer (pH 9.5) and incubated overnight at 4°C. The plates were washed with PBS-0.05% Tween-20 (PBST) and then blocked with PBS-1% bovine serum albumin (BSA, Sigma-Aldrich, USA) for 2 h at 37°C. After washing with PBST, 100 μL of the samples serially diluted with PBS-0.1%BSA was added, and the plates were incubated for 2 h at 37°C. After washing with PBST, an HRP-conjugated goat anti-mouse IgG or IgA antibody (Sigma-Aldrich, USA) was added at a 1:5000 dilution and incubated overnight at 4°C. A color reaction was developed using tetra-methyl benzidine substrate solution (Sigma-Aldrich, USA) at 37°C for 1 h. The reaction was stopped by adding 0.05 mL of 0.5 M H_2_SO_4_ per well, and the optical densities (ODs) were read at 450 nm using a microplate reader (Thermo Scientific Multiskan GO, USA). The antibody titers were expressed as the reciprocal of the highest dilution of sample for which the OD was 5×OD_mean background_, and the geometric mean titer (GMT) was calculated.

### Hemagglutination inhibition (HI) antibody titers against trivalent influenza virus antigens

To determine the HI antibody titers, the sera were treated with receptor-destroying enzyme (RDE, Denka Seiken, Tokyo, Japan) by incubation overnight at 37°C and then heated at 56°C for 50 minutes to deactivate the RDE. The sera were serially diluted (1:2), mixed with 4 HA units of influenza virus antigen (A/H1N1, A/H3N2 or B), and incubated at room temperature (RT) for 1 h. Finally, 1% chicken red blood cells were added, and the mixture was incubated at RT for 30 minutes. The HI antibody titers were defined as the highest dilution that completely inhibited hemagglutination.

### *In vitro* splenic lymphocyte stimulation and cytokine release

The spleens were harvested on day 21, and splenic lymphocyte suspensions were isolated using a mouse splenic lymphocyte separation solution kit (TBD Science Co., Ltd., Tianjin, China). The cells were adjusted to a concentration of 2 × 10^6^ cells/mL and cultured with ITIV antigens at a final concentration of 40 μg/mL of HA in 96-well plates. Wells without antigen were set as the background control. After 72 h of stimulation, splenic lymphocyte proliferation was measured using an MTT assay. The splenic lymphocyte proliferative rate (%) was calculated according to the following formula: the splenic lymphocyte proliferative rate (%) = (the mean OD_ITIV treatment group_—the mean OD_control group_)/the mean OD_control group_. The cell culture supernatants were collected by centrifugation and stored at −70°C for IFN-γ cytokine analysis using an ELISA kit (BioLegend, San Diego, CA, USA).

### Safety evaluation of the PA-adjuvanted influenza vaccine delivered by intranasal administration

Mice (6 males and 6 females per group) were administered the unadjuvanted ITIV or the PA-adjuvanted ITIV intranasally on days 0, 7, and 14 in three doses. After the first vaccination, the general status and local symptoms of irritation in the nose of each mouse were assessed every day, and the body weight of each mouse was examined on days 0, 7, 14, and 21. Half of the mice in each group were sacrificed at 24 h or 7 days after the third vaccination. The nose, lungs, spleen, liver, heart, and kidneys were harvested, and the gross morphology of these organs, including the color, texture, and size was observed immediately by double-blinded pathologists. All tissue samples were fixed in 4% formaldehyde for 24 h at 4°C, dehydrated by gradual soaking in alcohol and xylene, and finally embedded in paraffin. The paraffin-embedded specimens were cut into 5-μm sections and stained with HE. All sections were observed by double-blinded pathologists, and histological images were taken using an Olympus BX51 microscope with the DP71 system.

### Statistical analysis

All values are expressed as the mean values ± SE or SD. Fisher's exact test was used to compare the differences between each group for the seroprotection rate or seroconversion rate, mucosal response, and total response. One-way ANOVA with Tukey’s test was used to determine the significance of differences for multiple comparisons of other data. The analysis was performed in the GraphPad Prism 5 software. Differences with a probability value of *P* < 0.05 were considered significant.

## Results

### Antigen-specific serum or nasal and trachea wash antibody levels

**Fig 1:**
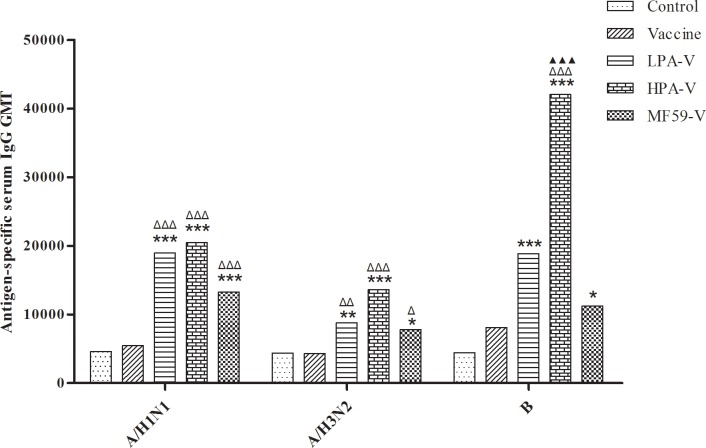
Antigen-specific serum IgG GMTs in the different groups. Mice were administered PBS (Control), ITIV (0.5 μg A/H1N, A/H3N2, and B HA/mouse; Vaccine), ITIV plus PA (500 μg/mouse; LPA-V or 1000 μg/mouse; HPA-V) or MF59 (5 μL/mouse; MF59-V) intranasally once a week for 3 weeks. Blood plasma was prepared from the mice 1 week after the final vaccination, and the antigen-specific IgG levels were determined by ELISA. For each ITIV strain, three serial dilutions were prepared for each serum sample in duplicate. A curve with the diluted factor on the X-axis and the optical densities (ODs) on the Y-axis and the associated linear equation were obtained. Based on the linear equation, the antigen-specific serum IgG antibody titer of each sample was calculated when the OD was 5×OD_mean background_. Finally, the geometric mean titer (GMT) was calculated. The data are shown as the GMT ± SE (n = 12). *P<0.05, **P<0.01, ***P<0.001 *vs*. Control group; ^Δ^*P*<0.05, ^ΔΔ^*P*<0.01, ^ΔΔΔ^*P*<0.001 *vs*. Vaccine group; ^▲▲▲^*P*<0.001 *vs*. MF59-V group.

**Fig 2:**
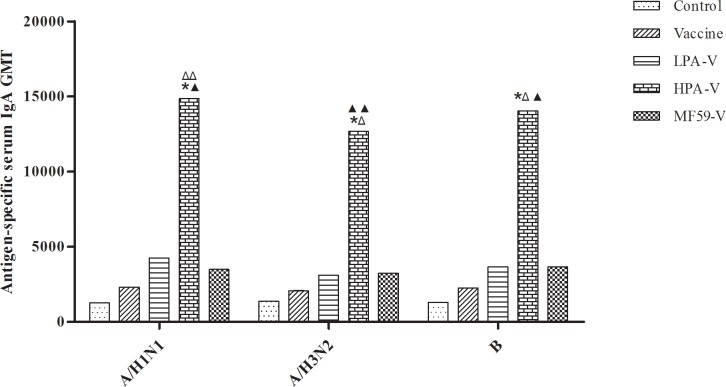
Antigen-specific serum IgA GMTs in the different groups. The mice received intranasal administrations as described in Fig 1. The serum antigen-specific IgA levels were determined by ELISA as described in Fig 1 and presented as the GMT ± SE (n = 12). *P<0.001 *vs*. Control or Vaccine group; ^▲^*P*<0.05, ^▲▲^*P*<0.01 *vs*. LPA-V group; ^Δ^*P*<0.05, ^ΔΔ^*P*<0.01 *vs*. MF59-V group.

**Fig 3:**
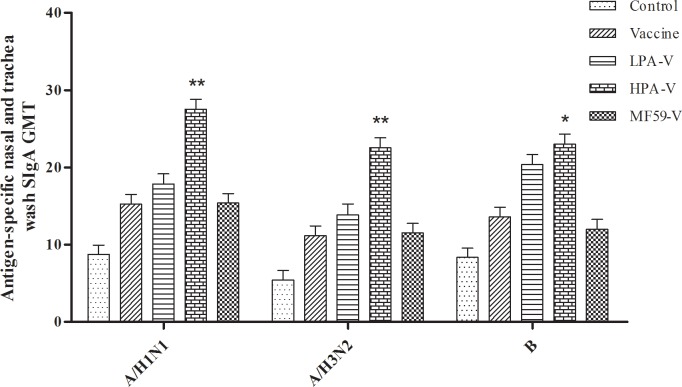
Antigen-specific nasal and trachea wash SIgA GMTs in the different groups. The mice received intranasal administrations as described in Fig 1. The antigen-specific nasal and wash SIgA levels were determined by ELISA as described in Fig 1 and presented as the GMT ± SE (n = 12). *P< 0.05, **P< 0.01 *vs*. Control group or Vaccine group.

As shown in Fig 1, the GMTs of antigen-specific serum IgG were significantly increased in both the LPA-V and HPA-V groups compared to the control group (*P*<0.001 or 0.01), particularly in the HPA-V group, but not in the vaccine group (*P*>0.05), although the levels were slightly increased in this latter group. The GMTs of antigen-specific serum IgG against the A/NYMCX-179A (A/H1N1) and A/IVR-165 (A/H3N2) strains were also significantly higher in the two PA-adjuvanted groups than in the vaccine group (*P*<0.001 or 0.01), whereas the GMT of the B/NYMCBX-39 (B) antigen-specific serum IgG was significantly enhanced in the HPA-V group (*P*<0.001). However, no significant differences were found between the PA-adjuvanted groups (*P*>0.05). In contrast to the serum IgG GMT, the GMTs of antigen-specific serum IgA were increased in all three vaccine immunization groups with or without the PA adjuvant, although significance was only reached between the HPA-V group and the other groups (*P*<0.05, 0.01 or 0.001, Fig 2). The GMTs of the antigen-specific nasal and trachea wash SIgA were similar to the GMTs of the antigen-specific serum IgA, but no significant difference was detected between the HPA-V group and the LPA-V group (*P*>0.05, Fig 3). These results demonstrated that the novel adjuvant PA significantly improved both the systemic and local mucosal immune responses to the ITIV elicited by intranasal vaccination and that its adjuvant effect was better in the HPA-V group.

### Hemagglutination inhibition (HI) antibody titers against trivalent influenza virus antigens

The serum HI GMTs against the ITIV antigens are shown in Table 2. The pre-vaccination serum HI GMTs against the ITIV antigens were comparable among the different groups (*P*>0.05). However, consistent with the serum IgG GMTs, the post-vaccination serum HI GMTs against the A/H1N1 and A/H3N2 strains and their fold increases compared to the pre-vaccination levels were significantly enhanced in the two PA-adjuvanted vaccine groups compared to the control group or the vaccine group (*P*<0.05, 0.01 or 0.001). However, in contrast to the serum B antigen-specific IgG GMTs, the serum HI GMTs against B antigen was not significantly increased in the two PA-adjuvanted groups (*P*>0.05, Table 2). Moreover, the serum HI GMTs against influenza virus antigens were not significantly different between the PA-adjuvanted vaccine groups (*P*>0.05, Table 2).

**Table 2:** Serum HI GMTs following intranasal immunization in the different groups.

Groups	Pre-vaccination (95% CI)	Post-vaccination (95% CI)	Fold increases
**Control**			
**A/H1N1**	5.25 (3.71–7.14)	4.57 (2.29–9.12)	0.87
**A/H3N2**	3.72 (2.41–5.73)	2.29 (1.68–3.13)	0.62
**B**	4.64 (2.45–8.79)	3.98 (2.34–6.77)	0.86
**Vaccine**			
**A/H1N1**	5.25 (3.26–8.46)	5.27 (2.11–13.15)	1.00
**A/H3N2**	5.12 (4.05–6.47)	5.46 (2.48–12.02)	1.07
**B**	3.98 (2.50–6.34)	5.26 (2.80–9.87)	1.32
**LPA-V**			
**A/H1N1**	5.01 (3.16–7.94)	20.28 (9.97–41.21) *^▼^	4.05**^▼▼^
**A/H3N2**	4.57 (2.90–7.20)	21.13 (10.35–43.15) ***^▼^	4.62**^▼▼^
**B**	4.64 (2.98–7.22)	5.84 (3.41–10.00)	1.26
**HPA-V**			
**A/H1N1**	5.12 (3.74–7.00)	20.65 (10.54–40.36) **^▼^	4.04**^▼▼^
**A/H3N2**	4.81 (3.56–6.49)	28.38 (14.26–56.49) ***^▼▼^	5.90**^▼^
**B**	4.57 (3.34–6.25)	6.92 (5.06–9.46)	1.51
**MF59 -V**			
**A/H1N1**	5.62 (4.33–7.30)	21.29 (13.80–32.21) **^▼^	3.75*^▼^
**A/H3N2**	4.90 (3.26–7.36)	27.99 (12.94–60.53) ***^▼▼^	5.71**^▼^
**B**	5.25 (3.71–7.41)	13.87 (8.76–21.93) **^▼^	2.64

* *P*<0.05

** *P*<0.01

*** *P*<0.001 *vs*. Control group

^▼^*P*<0.05

^▼▼^*P*<0.01

^▼▼▼^*P*<0.001 *vs*. Vaccine group.

GMT fold increases were defined as the GMT ratio of the post-vaccination titer to the pre-vaccination titer.

### Antibody responses after intranasal vaccination

According to their definitions [22], the seroprotection rate, seroconversion rate, and mucosal immune response were calculated and are shown in Table 3. The seroprotection rates and seroconversion rates were increased in both PA-adjuvanted groups, particularly for the A/H1N1 and A/H3N2 antigens, with a significant difference for the A/H3N2 antigen observed between the HPA-V and vaccine groups (*P*<0.05). In addition to the systemic responses, the mucosal immune responses were obviously increased in the vaccine group and both PA-adjuvanted groups, and significance was reached not only in the HPA-V group for the A/H1N1 and A/H3N2 strains (*P*<0.05 or 0.001) but also in both PA-adjuvanted groups for the B strain (*P*<0.05 or 0.001) compared to the control group. Additionally, the mucosal immune response to the A/H3N2 strain in the HPA-V group was superior to the response obtained in the vaccine group and the LPA-V group (*P*<0.05). The total responses were similar to the mucosal immune response, but the total responses for the A/H3N2 strain were also significantly higher in the LPA-V group compared to the control group (*P*<0.05).

**Table 3:** Antibody responses following intranasal immunization in the different groups.

Groups	Seroprotection rate^a^		Seroconversion rate^b^		Mucosal response^c^		Total response^d^	
	%	n/N	%	n/N	%	n/N	%	n/N
**Control**								
**A/H1N1**	0.00	0/10	0.00	0/10	16.67	2/12	16.67	2/12
**A/H3N2**	0.00	0/10	0.00	0/10	16.67	2/12	16.67	2/12
**B**	0.00	0/9	0.00	0/9	8.33	1/12	8.33	1/12
**Vaccine**								
**A/H1N1**	0.00	0/9	0.00	0/9	58.33	7/12	58.33	7/12
**A/H3N2**	0.00	0/11	0.00	0/11	58.33	7/12	58.33	7/12
**B**	0.00	0/10	0.00	0/10	50.00	6/12	50.00	6/12
**LPA-V**								
**A/H1N1**	9.09	1/11	9.09	1/11	40.00	4/10	40.00	4/10
**A/H3N2**	20.00	2/10	20.00	2/10	60.00	6/10	70.00^▼^	7/10
**B**	0.00	0/9	0.00	0/9	70.00^▼▼^	7/10	70.00^▼▼^	7/10
**HPA-V**								
**A/H1N1**	18.18	2/11	18.18	2/11	63.64^▼^	7/11	63.64^▼^	7/11
**A/H3N2**	40.00*	4/10	40.00*	4/10	100.00^▼▼▼^*^†^	11/11	100.00^▼▼▼^*	11/11
**B**	0.00	0/10	0.00	0/10	54.55^▼^	6/11	54.55^▼^	6/11
**MF59 -V**								
**A/H1N1**	10.00	1/10	10.00	1/10	63.64^▼^	7/11	60.00	6/10
**A/H3N2**	36.36	4/11	36.36	4/11	63.64^▼^	7/11	90.91^▼▼▼^	10/11
**B**	0.00	0/10	0.00	0/10	63.64^▼▼^	7/11	63.64^▼▼^	7/11

^a^ Seroprotection rate defined as the proportion of mice with a titer level ≥1:40 post-vaccination.

^b^ Seroconversion rate defined as the proportion of mice with a ≥4-fold increase in titer from baseline or a post-vaccination titer level ≥1:40 if the baseline titer was <1:40.

^c^ Mucosal response defined as the proportion of mice with a 1.4-fold increase in the SIgA titer compared to the mean titer of the control group.

^d^ Total response in reference to any response (mucosal and/or serum antibody response) following intranasal immunization.

* *P*<0.05 *vs*. Vaccine group

^▼^*P*<0.05

^▼▼^*P*<0.01

^▼▼▼^*P*<0.001 *vs*. Control group

^† ^*P*<0.05 *vs*. LPA-V group.

### Cellular immune response after intranasal vaccination

**Fig 4:**
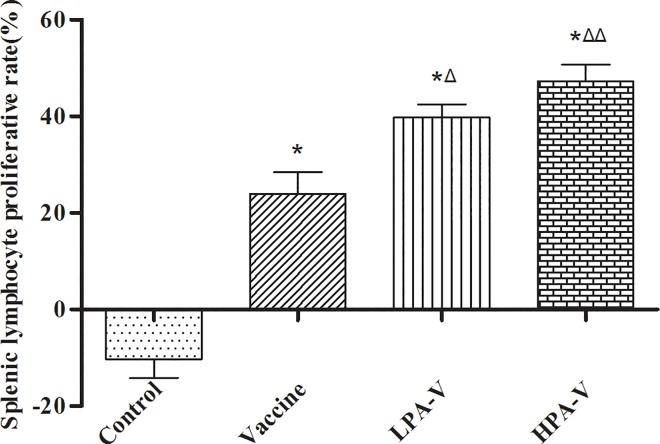
Splenic lymphocyte proliferative rates of the different groups after 72 h of culture under stimulation with three influenza virus antigens. The mice received intranasal administrations as described in Fig 1. The spleens were harvested on day 21, and splenic lymphocyte were cultured with a final concentration of 40 μg/mL of HA or without ITIV antigens. After 72 h, splenic lymphocyte proliferation was measured using the MTT assay. The splenic lymphocyte proliferative rate (%) was calculated according to the following formula: the splenic lymphocyte proliferative rate (%) = (the mean OD_ITIV treatment group_—the mean OD_control group_)/the mean OD_control group_ (n = 12). *P<0.001 *vs*. Control group; ^Δ^*P*<0.05, ^ΔΔ^*P*<0.01 *vs*. Vaccine group.

**Fig 5:**
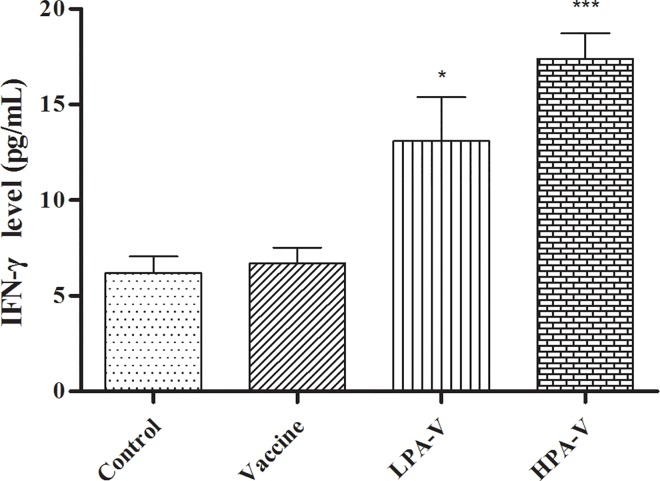
IFN-γ levels in the splenic lymphocyte supernatants induced by stimulation with three influenza virus antigens after 72 h in culture. After culture for 72 h with a final concentration of 40 μg/mL of HA or without ITIV antigens, the splenic lymphocyte supernatants were collected by centrifugation. According to the ELISA kit manufacturer's instructions, 50 μL of the cell supernatant was added to each well of the plate for IFN-γ cytokine analysis (n = 5). *P<0.05, ***P<0.001 *vs*. Control or Vaccine group.

After 72 h of stimulation with the ITIV antigens, the splenic lymphocyte proliferative rates and IFN-γ levels were significantly increased in the LPA-V and HPA-V groups compared to the corresponding values obtained in the control and vaccine groups (*P*<0.01, 0.05 or 0.001, Figs 4 and 5). These results indicated that PA successfully enhanced the cellular immunity promoted by intranasal ITIV vaccination.

### Adjuvant effect comparison of PA and MF59 for the ITIV

The MF59 adjuvant was used to further analyze the adjuvant effect of PA for the ITIV in this study. The results showed that MF59 significantly improved the antigen-specific serum IgG GMTs and HI GMTs for the A/H1N1 and A/H3N2 antigens, which were similar to the GMTs obtained for the LPA-V and HPA-V groups (Fig 1 and Table 2). Although the B antigen-specific serum IgG GMT in the MF59 group was significantly lower than the value obtained for the HPA-V group (*P*<0.001), the B antigen-specific serum HI GMT in the MF59 group was better than the value obtained in the HPA-V group and was significantly higher than the value obtained in the vaccine group (*P*<0.05) (Table 2). Moreover, by enhancing the antigen-specific serum IgA GMT and the mucosal SIgA GMT, the adjuvant effect of MF59 was likely lower than the adjuvant effect of the high-dose PA (Figs 2 and 3). However, the antibody response, including the seroprotection rate or seroconversion rate, the mucosal response, and the total response, was comparable between the HPA-V and MF59-V groups (*P*>0.05, Table 3).

### Safety evaluation of the intranasal administration of the PA-adjuvanted influenza vaccine

**Fig 6:**
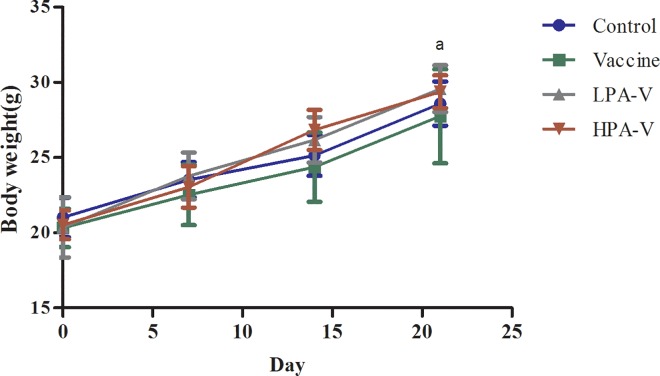
The body weights of the mice in the different groups. After the first vaccination, the body weight of each mouse was examined on days 0, 7, 14, and 21. The mean body weight of the mice in each group was calculated at the indicated time point (mean ± SD, n = 12; ^a^n = 6).

**Fig 7:**
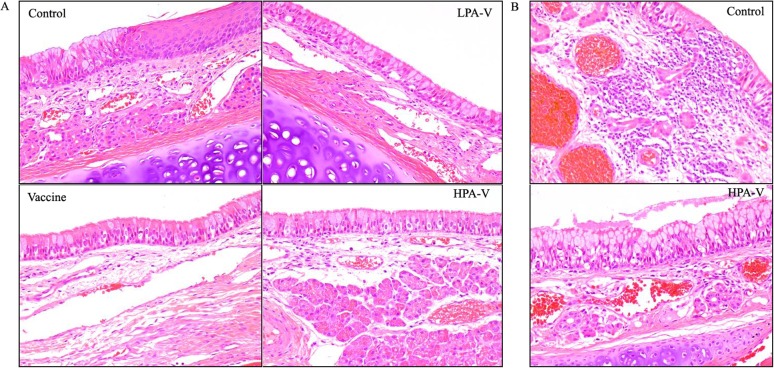
The nose mucosa histopathology of the mice in the different groups on day 15. After three intranasal immunizations at 1-week intervals, the nose tissues of the mice were examined by light microscopy on day 15 or day 21. Representative histopathology of the HE-stained nose tissue on day 15 is shown. A: Normal structure of the nose mucosa in the different groups; B: Hyperemia with the infiltration of a few inflammatory cells in the lamina propria of the nose of one mouse from the control and HPA-V groups.

No abnormal signs were observed in the mice during the experiment. The body weights of the mice were not significantly different between any of the groups at each time point (Fig 6). Furthermore, the gross morphology of all organs on days 15 and 21 was normal. Histologically, the nose tissues were normal in most of the mice in the different groups (Fig 7A) on day 15; however, hyperemia with the infiltration of a few inflammatory cells was observed in the lamina propria of the nose in one mouse from the control group and one mouse from the HPA-V group (Fig 7B). No obvious changes in other tissues, including the lungs. Furthermore, no pathological changes were observed in any of the examined tissues from all mice on day 21.

## Discussion

PA has been reported to modulate immune functions via various pathways, which may activate T and B lymphocytes and NK cells to produce interferons and interleukin cytokines or antibodies and mediate an increase in the CD4^+^ T-cell count and CD4^+^/CD8^+^ ratio [23, 24]. Recently, Liu et al. showed that PA improved the production of anti-HBs antibodies after hepatitis B virus vaccine immunization [18]. Therefore, we hypothesized that PA might be an ideal potential adjuvant for vaccines. Based on our previous studies on intranasal immunization with PA-adjuvanted H1N1 and EV71 vaccines [19, 20], we speculated that PA might be an ideal novel mucosal adjuvant for intranasal administration of an influenza vaccine.

In this study, we explored the adjuvant effect of PA on the immunogenicity of the ITIV using intranasal vaccination in mice. The results showed that the PA adjuvant significantly increased the systemic and local antibody titers and the cellular immune response to the ITIV, particularly in the HPA-V group. The HPA-V group nearly fulfilled the immunogenicity evaluation criteria of the Committee for Medical Products for Human Use (seroprotection rate >60%, seroconversion rate >30%, and GMT fold >2) [1], with a seroconversion rate for the A/H3N2 antigen of 40% and fold increases in the HI GMTs for the A/H1N1 and A/H3N2 antigens of 4.04 and 5.90, respectively. Similarly, the mucosal antibody response and the total response were significantly improved in the HPA-V group, particularly for the A/H3N2 antigen. Additionally, high-dose PA was comparable to the MF59 adjuvant in improving the humoral immune response of the ITIV, particularly for the influenza virus A/H1N1 and A/H3N2 antigens, but was better at inducing a mucosal immune response to the ITIV.

Currently, serum HI titers are used to evaluate the efficacy of influenza vaccines [25]. However, both serum and nasal antibody responses are involved in protection, and together they might result in better correlates for protection against heterologous influenza strains [26, 27]. Additionally, the SIgA antibodies in the upper respiratory tract are considered the first line of defense to prevent viral infections and are more cross-reactive against variant influenza viruses than serum IgG antibodies; therefore, SIgA antibodies provide more effective protection against a heterologous virus [28, 29]. Moreover, serum IgA acts as a second line of defense by eliminating pathogens that have breached the mucosa [30]. In the present study, the mucosal SIgA GMTs and the serum IgA levels in the HPA-V group were not only significantly improved but also significantly higher than the values obtained by the intramuscular injection of the vaccine alone, although the antigen-specific serum IgG and HI GMTs were higher (data not shown). Therefore, we reasoned that the mice in the HPA-V group might achieve effective protection against influenza viruses. We confirmed that intranasal vaccination was better than intramuscular vaccination, particularly in inducing the mucosal immune response against the ITIV. However, we believe that the serum IgA or mucosal SIgA levels should be used for the evaluation of the efficacy of intranasal vaccination with influenza vaccines in addition to the serum HI titers to determine whether the mice in the HPA-V group truly gained protective efficacy against the influenza virus. This possibility needs to be explored in the future.

Studies have reported that cell-mediated immunity is related to the development of immunity against influenza infection and have revealed that the immunogenicity of influenza vaccines may be evaluated by IFN-γ production against the vaccine antigen [31–33]. In the present study, PA significantly elevated IFN-γ production by splenic lymphocytes after vaccine antigen stimulation, which indicated that PA could promote the cell-mediated immune response after intranasal ITIV vaccination. A shift from Th1 cytokines (including IFN-γ) to Th2 cytokines (including IL-10) with aging has been associated with reduced cytotoxic T lymphocyte activity and diminished protection against influenza virus challenge [34], and age-related susceptibility to influenza can be reversed by the induction of a more potent IFN-γ response [35–37]. Therefore, the influenza vaccine needs to more effectively stimulate IFN-γ in elderly individuals. A/H3N2 strains are the most virulent strains in elderly adults, followed by B strains [38, 39]. In the present study, in addition to significantly elevating IFN-γ production by splenic lymphocytes, high-dose PA excellently improved the humoral immune response against influenza virus H3N2 strains in young mice. Thus, we infer that intranasal immunization using a PA-adjuvanted influenza vaccine may be more suitable for older adults. However, this hypothesis requires further confirmation.

The MF59 adjuvant, which is an oil-in-water emulsion, provides immunogenicity and protection against the influenza virus in humans and is the most commonly used adjuvant for influenza virus vaccines [2, 40]. Therefore, the MF59 adjuvant was compared with the PA adjuvant in this study. In agreement with previous reports [35, 40–42], the results showed that the antigen-specific serum IgG GMTs and post-vaccination HI GMTs for both the A/H1N1 and A/H3N2 strains were significantly improved by the MF59 adjuvant and were significantly higher than the GMTs obtained with the vaccine alone. These effects were observed in the two PA-adjuvanted vaccine groups, although the serum IgA and mucosal SIgA GMTs were significantly enhanced by the high-dose PA adjuvant compared to the MF59 adjuvant. Additionally, the MF59 adjuvant was better than the high-dose PA adjuvant at promoting the antibody response to the B antigen. Thus, we believe that the high-dose PA adjuvant is comparable with the MF59 adjuvant in improving the humoral immune response to the ITIV, particularly for influenza virus A/H1N1 and A/H3N2 antigens, but is better at inducing the mucosal immune response to the ITIV.

Concerns have been raised about the potential side effects induced by intranasal vaccination with inactivated influenza virus antigens, particularly with regard to the possibility of nerve cell damage and Bell's palsy due to the cholera toxin or *Escherichia coli* heat-labile toxin adjuvant [15, 29]. Although PA is widely and safely used as an immunomodulator in the clinic, the safety of the PA-adjuvanted inactivated influenza vaccine is unclear. Thus, we systemically explored the side effects of intranasal immunization of the PA-adjuvanted inactivated influenza vaccine in mice. The results showed that two different doses of PA-adjuvanted vaccines were safe, without any abnormal signs or pathological changes in the nasal mucosa, lungs, liver, heart, spleen or kidneys.

This study has the following limitations: (1) the long term immunity of the PA-adjuvanted vaccine was unclear, although both the serum and mucosal antibody levels, as well as the splenic lymphocyte proliferation and IFN-γ level, were improved at day 21 after intranasal immunization with the high-dose PA-adjuvanted vaccine, and (2) we did not evaluate whether the immunity induced by intranasal vaccination with the PA-adjuvanted vaccine could protect the mice from lethal influenza virus challenge. These issues will be explored in future research.

## Conclusions

This study was the first to explore the adjuvant effect and safety of PA for intranasal vaccination with the ITIV. The results support our hypothesis that PA is a novel mucosal adjuvant for intranasal vaccination with the ITIV, with safe and effective mucosal adjuvanticity in mice. The PA-adjuvanted vaccine successfully induced both serum and mucosal antibody responses and a cell-mediated response, and the efficacy of high-dose PA was comparable to that of the MF59 adjuvant. Although virus challenge of the mice after intranasal vaccination was not evaluated in the present study and the mechanism of the adjuvant effect of PA was unclear, this study may help further develop the intranasal PA-adjuvanted ITIV and accumulate evidence for the application of PA as a novel adjuvant in other vaccines.
